# Supplementary material for: Genetic Reassortment between Endemic and Introduced Macrobrachium rosenbergii Nodaviruses in the Murray-Darling Basin, Australia
Source: Viruses. 2022 Oct 4;14(10):2186. doi: 10.3390/v14102186 (PMC9612130; doi:10.3390/v14102186)
Supplement: Supplementary file 1 [file viruses-14-02186-s001.zip › viruses-1952632-supplementary/SiFigureS2_Capsid_alignment.pdf]

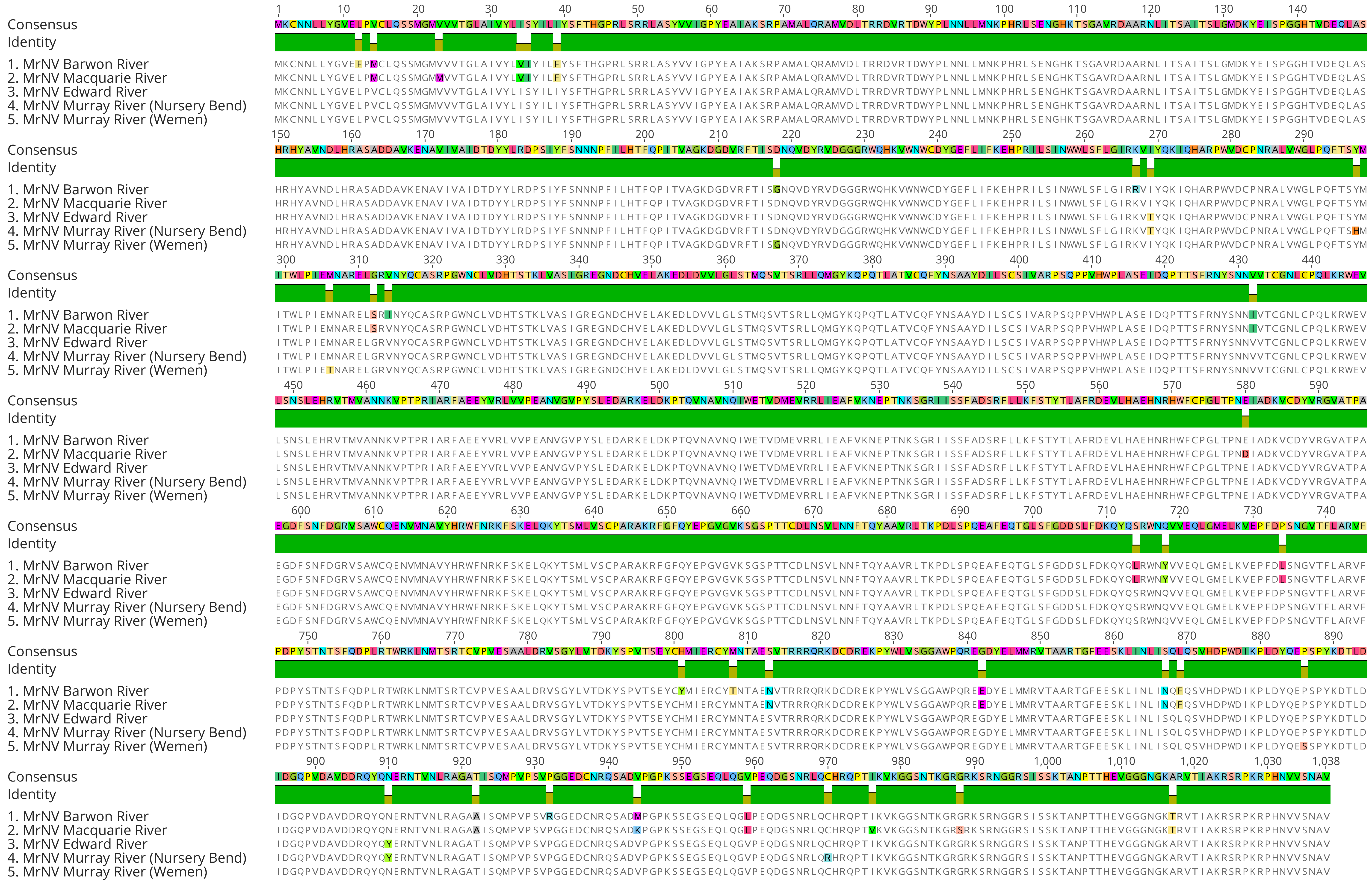
Consensus
Identity
1. MrNV Barwon River
2. MrNV Macquarie River
3. MrNV Edward River
4. MrNV Murray River (Nursery Bend)
5. MrNV Murray River (Wemen)
